# Factors associated with resilience among medical residents in Tunisia

**DOI:** 10.1192/j.eurpsy.2025.2015

**Published:** 2025-08-26

**Authors:** H. Daoud, I. Sellami, A. Feki, M. Hajjaji, M. L. Masmoudi, K. Jmal Hammami

**Affiliations:** 1 Department of Occupational Medicine, Hedi Chaker Hospital; 2 LR/18/ES-28, Sfax university; 3 Department of rheumatology, Hedi Chaker Hospital, Sfax, Tunisia

## Abstract

**Introduction:**

Resilience plays an essential role in many settings, including medical resident training. This group undergoes a unique educational process that demands the development of both clinical and interpersonal skills, as well as a high level of personal responsibility for patient care.

**Objectives:**

The aim of this study was to evaluate resilience among medical residents and identify the factors that influence it.

**Methods:**

We conducted a cross-sectional study involving medical residents in training at Tunisian hospitals. Data were collected anonymously through a questionnaire-based survey using Google Forms between October 2023 and January 2024. Participants completed a sociodemographic form, followed by two scales: the Brief Resilience Scale (BRS) to measure resilience and the Perceived Stress Scale (PSS-4) to assess mental health disorders.

**Results:**

Our study included 127 participants, with the majority being women (74.8%). The average age was 27.24 ± 1.34 years. Most participants were single (81.8%), and only 5.5% were international residents. Approximately one-third (37%) engaged in physical activity. A psychiatric history was reported in 17.3% of the residents. The mean Brief Resilience Scale (BRS) score was 3.167 ± 0.77. Low resilience scores were observed in 34.6% of participants, while only 6.3% had high resilience scores. The mean Perceived Stress Scale (PSS-4) score was 7.08 ± 2.7. Univariate analysis revealed that lower resilience levels were more common among females (p = 0.000) and residents with a history of psychiatric disorders (p = 0.06). Additionally, lower resilience levels were associated with higher stress levels (p = 0.00; r = -0.512).

**Conclusions:**

The study highlights the need for targeted interventions to enhance resilience among medical residents, particularly for those in vulnerable subgroups. Improving resilience through tailored support strategies can significantly benefit their overall well-being and training outcomes.

**Disclosure of Interest:**

None Declared

